# Biosensors-on-Chip: An Up-to-Date Review

**DOI:** 10.3390/molecules25246013

**Published:** 2020-12-18

**Authors:** Cristina Chircov, Alexandra Cătălina Bîrcă, Alexandru Mihai Grumezescu, Ecaterina Andronescu

**Affiliations:** 1 Department of Science and Engineering of Oxide Materials and Nanomaterials, University Politehnica of Bucharest, 011061 Bucharest, Romania; cristina.chircov@yahoo.com (C.C.); ada_birca@yahoo.com (A.C.B.); ecaterina.andronescu@upb.ro (E.A.); 2 Research Institute of the University of Bucharest—ICUB, University of Bucharest, 050657 Bucharest, Romania

**Keywords:** biosensors on a chip, biosensors, microfluidics, point-of-care, diagnostics

## Abstract

Generally, biosensors are designed to translate physical, chemical, or biological events into measurable signals, thus offering qualitative and/or quantitative information regarding the target analytes. While the biosensor field has received considerable scientific interest, integrating this technology with microfluidics could further bring significant improvements in terms of sensitivity and specificity, resolution, automation, throughput, reproducibility, reliability, and accuracy. In this manner, biosensors-on-chip (BoC) could represent the bridging gap between diagnostics in central laboratories and diagnostics at the patient bedside, bringing substantial advancements in point-of-care (PoC) diagnostic applications. In this context, the aim of this manuscript is to provide an up-to-date overview of BoC system development and their most recent application towards the diagnosis of cancer, infectious diseases, and neurodegenerative disorders.

## 1. Introduction

**Figure 1:**
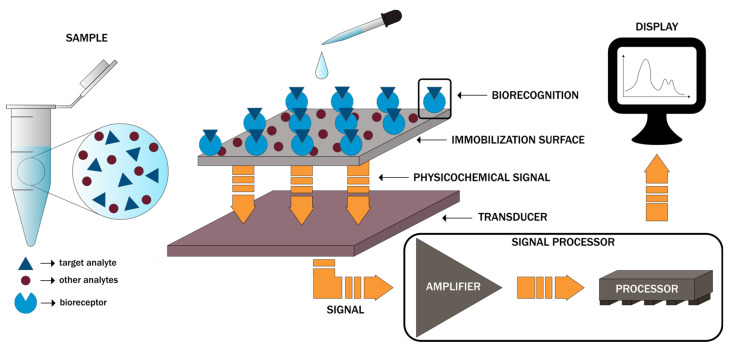
The main components of biosensors and the basic working principle.

As defined by the International Union of Pure and Applied Chemistry (IUPAC), a biosensor is “a self-contained integrated device which is capable of providing specific quantitative or semi-quantitative analytical instrumentation using a biological recognition element (biochemical receptor) which is in direct spatial contact with a transducer element”. Generally, biosensors are designed to translate physical, chemical, or biological events into measurable signals [1,2]. In this regard, there are three main components within a biosensor, namely the bioreceptor consisting of biomolecules, such as enzymes, proteins, nucleic acids, aptamers, antibodies, organelles, microorganisms, or cell receptors, responsible for the selectivity towards the target analyte, the transducer, such as optical, electrochemical, physicochemical, piezoelectric, mechanical, or thermal, that converts the biorecognition event proportional to the target analyte concentration into a quantifiable electrical signal, and the electronic system, comprising an amplifier, a processor, and a display unit, that will further process the signal into a user-friendly visualization (Figure 1) [3,4,5,6,7,8].

**Figure 2:**
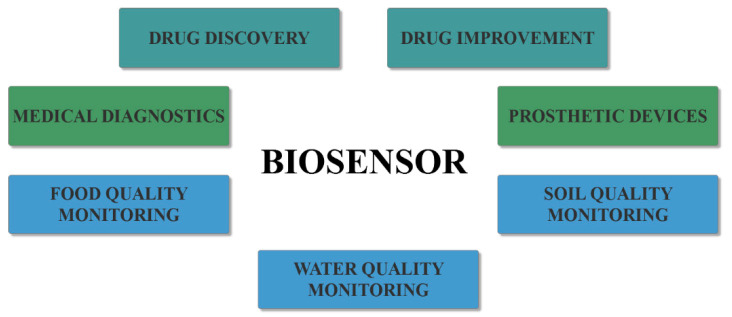
The main applications of biosensors.

The multidisciplinary nature of biosensors, involving biology, physics, chemistry, electronics, instrumentation, and economics, has led to an alliance among the experts within the different fields for bridging the gap between academic research and commercially viable products [6,9,10]. Therefore, biosensors have been developed for a wide variety of applications within the medical, environmental, pharmaceutical, and food fields (Figure 2) [5], namely for drug improvement, nutrition safety by detecting drugs and toxins in food, or ecology measuring and monitoring by detecting pollutants, microorganisms, or hazardous chemicals in water or soil [6,11]. However, the biosensor arena is rapidly expanding, especially within the healthcare system, because of their immense potential for medical diagnostics [11,12,13,14].

While any biosensor is characterized by several advantages and disadvantages based on the targeted application, their design generally involves high ligand specificity and selectivity, high-throughput capacity, dynamic range, rapid detection, ease of engineering and operating, cost-efficiency, and low power requirements [14,15,16]. However, further improvements for developing ultrasensitive assays capable of single-molecule detection are required [17,18,19]. In this regard, combining biosensor technologies with microfluidics and nanotechnology could offer the means for the accurate and timely diagnosis of various diseases [20]. Microfluidics, which aims to spatially and temporally control fluids within microscaled systems [21], is the research field with significant applicability in quantitative and qualitative analyses of biological and chemical entities [22,23]. Through its capacity of accurately manipulating small volumes of samples and its highly controlled environments, molecules within the sample rapidly achieve measurable concentrations, thus enabling rapid detection and scaling through parallelization, reduced costs due to lower sample, reagent, and power consumption, and high processing [22,23,24,25]. Hence, by integrating biosensors with microfluidic technologies, biosensor-on-chip (BoC) systems with higher detection sensitivity and specificity, resolution, automation, throughput, reproducibility, reliability, and accuracy could be achieved [25,26,27,28,29,30,31,32,33]. Moreover, microfluidic platforms improve reagent mixing, as it provides reaction chambers for the loading and immobilization of biorecognition elements, thus allowing bioreaction initiation followed by the delivery to the transducer [30]. Thus, the advantages of BoC systems has led to substantial advancements in point-of-care (PoC) diagnostic applications [29], including cancer, a neurodegenerative disorder, and infectious disease diagnosis [34]. Such devices could bring significant improvements, especially in the resource-limited environment of the developing countries, as it has brought diagnostics out of central laboratories to the patient bedside [35,36,37].

In this context, the aim of this paper is to provide an overview of BoC system development and their most recent application towards the diagnosis of cancer, infectious diseases, and neurodegenerative disorders, thus emphasizing their potential as the future of medical diagnostics.

## 2. Design and Working Principles

BoC systems are based on micro-total analysis systems, which aim to provide complete analytical microscaled systems by diminishing and accumulating all steps required for sample analysis within a single device [26,38]. Specifically, it must allow the performance of standard laboratory functions, including sample injection, mixing, reaction, separation, and enrichment, and analyte detection [26,38,39,40]. Therefore, such systems may contain a variety of units, and their design is generally not straightforward [41,42], as they can function based on passive, active, or hybrid mechanisms. However, the experimental setup commonly involves four main modules: namely, the inlet unit, through which the sample is injected into the microchip; the reacting unit, where the required reactions occur; the analysis unit, where physicochemical reactions are detected by the biosensors; and the data processing unit, where the resulted signals are converted into output signals. Additionally, other modules, such as conditioning or amplification units, could also be introduced when necessary [42]. Furthermore, BoC systems should contain the main components necessary for fluid control and operation and chemical processes, such as microchannels, micropumps, microvalves, and micromixers [26,39,42,43].

Fluids are introduced into the microfluidic device via separate inlets and delivered into the microchannels, subsequently creating concentration gradients [27]. In this context, there are two types of inlets, namely vertical or top-loading inlets, where the loading tube is perpendicularly inserted, allowing for an easier fabrication due to increased compatibility with multilayered geometries, and parallel or in-line inlets, where the tube is parallelly introduced into the microchip [44].

Furthermore, micropumps and microvalves are key components for microfluidics-based analytical systems, especially in multi-step chemical reactions and quantitative analyses, as they enable complex biochemical assay automation [45,46]. On the one hand, micropumps are an integral part of microfluidic systems, as they allow for the precise, accurate, and reliable control of fluid transport through the device [45,47,48]. Moreover, they are capable of transferring fixed amounts of the fluid to the reaction unit for undergoing mixing, separation, or sensing processes [49]. The necessity of these micro-components has led to the development of various pumping techniques, which involve electrodes, valves, piezoelectric materials, or acoustic transducers introduction and consequently higher costs [48]. Additionally, since active micropumps require external energy supplies, which make them unsuitable for PoC applications, passive micropumps, which circumvent limitations related to power consumption, actuation mechanism integration, and pulsating or oscillating flows, are preferred for BoC development [47,48]. Among them, pneumatic-driven micropumps, which use compressed air for fluid driving, are the most commonly used [45]. On the other hand, microvalves are also fundamental for microfluidic systems, as their functions involve flow regulation, biomolecule, nanoparticle, or reagents isolation, and on/off switching [43,50]. General properties of microvalves include limited leakage, linear operation, small dead volume, insensitivity towards contamination, fast response, and low power consumption [43]. Their performance is directly influenced by structure and geometry, which should be designed for large-scale integration, minimized force for fluid control, portability, and energy efficiency [51]. Besides the general classification into active and passive microvalves, they can also be divided into normally open and normally closed [43]. Among them, the most widely used is the pressure-driven valve, where the membrane deformation leads to the pinch off or opening of the flow channel [46].

As they directly impact BoC efficiency and sensitivity, micromixers are one of the most fundamental components. By contrast to macroscaled fluidic devices, where fluid mixing occurs due to convection effects, microscale mixing generally involves external turbulences and/or microstructure integration [40]. Specifically, fluid flow within microfluidic devices is laminar with a Reynolds number lower than 1. Therefore, fluid flow rates are very low and in parallel layers, without disruption between layers. Consequently, fluid mixing depends on molecular diffusion, which is considerably inefficient [27,40]. In this manner, by integrating passive or active mixers, the slow mixing challenge can be overcome [27,52]. Passive micromixers are more economical and convenient, as the mixing occurs through molecular diffusion and chaotic advection with no external energy sources [52,53]. Chaotic advection is highly efficient at low Reynold numbers owing to the stream splitting, stretching, folding, or breaking up [54]. Moreover, it is generally achieved through microchannel geometry modifications in order to reduce diffusion length and increase interfacial area [27,53], subsequently causing pressure and velocity variations [54].

While simple microfluidic devices can be fabricated from any type of material, the properties of the selected materials will substantially impact the success of the device, as they directly influence its properties, overall performance, and applicability range [46,55]. Specifically, the material is involved in electrical conduction, heat transfer, light transmission, or mechanical transduction, and it influences the fluid physical behavior and constitution [55]. Additionally, the biocompatibility and wettability of the device are dependent upon the material choice and the microfabrication technique [41]. The most common materials used for microfluidic device development include glass, silicon, and thermoplastic or elastomeric polymers, such as polymethylmethacrylate (PMMA), polydimethylsiloxane (PDMS), and polycarbonate, owing to their cost-efficiency, biocompatibility, suitable physicochemical and mechanical properties, and ease of manufacturing [41,46,56].

However, recent years have witnessed a tremendous scientific interest in using paper as a material for microfluidic applications [57,58]. As cellulose is the basic component, the paper is a thin, light, and hydrophilic material, which allows for the spontaneous infiltration of water without requiring additional pumps [57]. Additionally, the paper is highly advantageous for BoC systems and PoC assays owing to its versatility, availability, and flexibility, allowing for the manufacture of portable, disposable, easy to operate, and cost-efficient devices [57,59,60]. Moreover, it can be chemically functionalized in order to design hydrophobic barriers for the spatially resolved transport of fluid [57,58]. However, there are still some issues related to the use of paper, such as the geometric and chemical complexity of the material and the imprecision associated with most paper patterning methods, which have considerably limited its applications [58,60].

**Figure 3:**
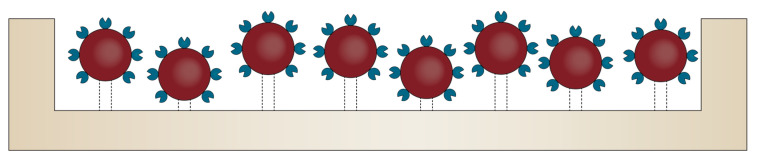
Schematic representation of the use of bioreceptor-functionalized nanomaterials deposited onto the surface of the microfluidic chip.

Nonetheless, biosensor performance is significantly influenced by the amount, spacing, and stability of the surface-immobilized bioreceptors within the BoC device [61]. Therefore, additional strategies for modifying their surface properties are often required [55]. In this context, after their surface functionalization with reactive functional groups, nanomaterials allow for the attachment of various biomolecules, such as antibodies, protein receptors, or peptides, in order to develop multifunctional materials for targeted treatment and diagnostics [62]. Owing to their small size and high surface areas, nanomaterials have revolutionized both the electrochemistry and electroanalysis fields, as they can be used for the detection of target analytes within extremely low-concentration solutions [62,63]. In addition, nanomaterials possess strong adsorption capacity and reduced non-specific adsorption due to abundant binding points, high conductivity, high passivation resistance, enhanced catalytic activity, and biocompatibility [20,61,63]. Therefore, the incorporation of nanomaterial-based biosensors within BoC systems has become a widely employed strategy for enhancing analytical performances (Figure 3) [61,63]. In this manner, the sensitivity, selectivity, stability, capture efficiency, and reproducibility of BoC are significantly improved [20,61,63,64]. However, the immobilization of the nanomaterial-based biosensors should allow for the proper orientation of the biorecognition molecules for target recognition and avoid its deterioration or leakage. Thus, atomic layer deposition is one of the most promising techniques used for the precise deposition of thin nanomaterial layers onto the surface, ensuring both conformality and uniformity for immobilization [20]. There have been many studies regarding the use of nanomaterials for BoC development, including carbon and metallic nanoparticles, nanobeads, nanotubes, nanowires, nanofibers, nanopillars, and nanohorns [20,63,64]. Additionally, graphene-based two-dimensional nanostructures have also attracted a great scientific interest in the recent years [63,64].

Furthermore, the combination of nanomaterials with microfluidics and modern optical sensing techniques has led to the emergence of a novel and rapidly growing interdisciplinary research field, namely optofluidics [65,66], which has significantly increased detection sensitivity and reduced the detection limit for various biosensors [67]. The surface-enhanced Raman spectroscopy (SERS) is an ideal example of a detection method that has considerably benefitted from the integration of optical nanosensors within microfluidic devices [68,69].

## 3. Diagnostic Applications

### 3.1. Cancer Diagnosis

Solid tissue biopsies remain the gold standard for tumor confirmation, diagnosis, and classification, consisting of tumor tissue removal and subsequent histopathological and cytological examination. Nevertheless, the procedure is highly painful and invasive. It further induces the risk of bleeding, inflammation, and malignant cell dissemination. Additionally, the amount of the extracted sample is generally insufficient. The spatial and temporal heterogeneity of the tumor tissue might lead to inconclusive results [70,71]. However, tumor tissues shed into the body fluids a variety of components, including cells, nucleic acids, proteins, or exosomes, which could serve as tumor biomarkers that provide similar information to tissue biopsies [70]. In this context, liquid or fluid biopsies have emerged as a term defining the set of diagnostic procedures using cancer-derived materials from body fluids [71,72]. This technique has provided a promising alternative to traditional biopsies due to the minimal invasiveness, cost-efficiency, and possibility of sampling at any time points during therapy to assess disease progression [70,71].

Although liquid biopsies can be sampled from any body fluid, blood is the most widely used as it allows for the detection of most cancer types, by contrast to urine or saliva, which are used for the detection of specific types. Nonetheless, the use of blood samples is still challenging due to the presence of countless cells and molecules, which makes the assessment of extremely low quantities of biomarkers difficult [71]. In this regard, the development of sensitive platforms able to routinely detect and quantify reduced levels of biomarkers within body fluids represents the means for enabling personalized medicine in cancer therapy [70]. Microfluidics-based technologies represent a promising solution in this field. There is a great scientific focus towards developing biosensor-integrated microfluidic chips for cancer diagnosis [70,71,73].

In this context, the process of paper selection for reviewing involved a literature survey using the Scopus database and the keywords “microfluidic (bio)sensor” and “cancer diagnosis”. A series of 17 potential papers published since 2018 related to the subject were identified. All papers are summarized in Table 1 and described in the following paragraphs.

Ever since their discovery, the detection of circulating tumor cells (CTCs) within liquid biopsies has attracted significant interest as a tool for early cancer diagnosis, metastatic cancer stage and cancer progression monitoring, and therapy response [92,93]. Microfluidic devices for CTCs analyses focus on cell isolation, detection, and examination, demonstrating a high efficiency, selectivity, and reactivity with reduced sample and reagent amounts and increased fluid control [71,92]. Most studies on the matter focus on isolating CTCs through immunoaffinity, which involves the immobilization of specific antibodies on the surface of the microchannels. Specifically, as it is overexpressed in most cancer types, the epithelial cell adhesion molecule (EpCAM) represents the target antigen in most assays [71,92]. However, strategies for isolating CTCs can also rely on the physical properties changes that occur due to abnormal metabolism and modifications of intracellular substance composition, gene expression, and protein synthesis and aggregation [71,94]. Thus, there are several microfluidic devices developed for the isolation of CTCs based on modifications within their size, density, morphology, stiffness, electrical charge, and dielectric properties [71,94,95,96]. Such label-free microfluidic methods include magnetic-, acoustic-, dielectrophoresis-, and passive microfluidic-based techniques [97]. Moreover, CTCs separation can be performed through positive or negative enrichment, involving the specific isolation of CTCs within the sample or the separation of all other cells except CTCs from the sample, respectively [71].

In this regard, Çetin et al. developed a gold microfluidic device consisting of a self-assembled monolayer formed onto the surface. The molecules used for the monolayer include cysteamine, 4-aminothiophenol, 3-mercaptopropionic acid, 11-amino-1-undecanethiol, and 11-mercaptoundecanoic acid, which had both amino and carboxylic free functional groups for the subsequent attachment of EpCAM antibodies through both covalent bonding via the carbodiimide crosslinking method and bioaffinity-based immobilization using streptavidin and biotinylated EpCAM. MCF-7 breast cancer cells were used as the CTCs model, while K562 leukemia cells were used as an EpCAM-negative model. Results showed that the presence of the aromatic ring in the within alkanethiols allowed for intermolecular interactions and consequently increased cell capture events. Additionally, covalent bonding permitted high flow rates within the microchannels, with negligible numbers of detached cells [74]. Furthermore, Chan et al. investigated the efficiency of PMMA microfluidic channels coated with EpCAM antibodies-immobilized polyoxazoline polymer through covalent bonds for the photodynamic identification of EJ138, HT1376, HT1197, and RT4 human bladder cancer cells treated with protoporphyrin IX. Their findings showed that hexaminolevulinate-induced fluorescence increased substantially in cancer cells, with optimal discrimination achieved for cells in suspension after incubation at 50 µM hexaminolevulinate for 2 h at 37 °C and the subsequent nuclear red treatment [75]. This study represents a continuation of their previous work for identifying cancer cells in urine [98]. Moreover, Nguyen et al. fabricated a microfluidic platform for the identification of A549 human lung carcinoma cells by combining DNA aptamers and electrical impedance measurements. Similarly, self-assembled monolayers containing carboxylic groups were grown onto the surface of gold electrodes for amino-labeled aptamer conjugation. Results demonstrated a high affinity towards target cells and increased detection sensitivity at the frequency of 5 kHz [76]. Another study performed by Anu Prathap et al. investigated the detection of circulating SK-MEL-2 human melanoma cells using an electrochemical immunosensor consisting of polyaniline nanofibers-modified electrodes with antibodies against the MC1R antigen. The sensor was able to selectively detect extremely low concentrations of 10 cells/10 mL of solution in the presence of peripheral blood mononuclear cells [77].

Although CTCs have been preferred in the microfluidic-based sensor research for cancer diagnosis, alternative biomarkers, including microRNAs (miRNAs), circulating tumor DNA, or cancer-related proteins, can also provide important clinical information in this regard [95,99].

In this context, Lunelli et al. comparatively investigated two PDMS microdevices with spiral-shaped configurations and different surface-to-volume ratios functionalized with positive charges for attracting the negative charges phosphate groups within the miRNA backbones. Results showed that a higher surface-to-volume ratio led to an increased miRNA capture, which is of significant importance considering the low concentrations of miRNA biomarkers within body fluids [78]. Additionally, Kutluk et al. compared the performance of competitive and sandwich assays in terms of selectivity, sensitivity, dynamic range, reproducibility, and handling, using miRNA-197 as the target biomarker in undiluted human serum samples. Based on their results, as sensitivity and selectivity are of priority, the sandwich assay might be more advantageous [79]. Moreover, Fakhri et al. employed a colorimetric strategy using a paper microfluidic device for the detection of miRNA-21 based on the peroxidase-like activity of single-stranded DNA attached to Ag/Pt nanoclusters. The device allowed for the quantitative measurement of miRNA, with a considerably low detection limit of 0.6 pM [80]. However, Tian et al. achieved a detection limit of 0.0033 fM for miRNA-21 using a lamellar MnO_2_ nanosheets-functionalized T-shaped duplex structure [81]. Another study investigated the efficiency of a clustered regularly interspaced short palindromic repeats (CRISPR)/Cas13a-powered microfluidic electrochemical biosensor for miRNA detection. Although the all-in-one BoC requires further improvements regarding sensitivity and reproducibility, it has proved to be an easy-to-use and efficient device with a limit of detection of 10 pM [82].

Veselinovic et al. further demonstrated that nanostructuring the surface of electrodes leads to higher surface coverage and consequently enhanced sensor performance. Specifically, they integrated nanoporous gold multielectrode arrays within microchannels for facilitating the multiplexed detection of DNA biomarkers for cancer, namely the BRCA1, BRCA2, and p53 breast cancer-related genes, through electrophoretic guidance [83]. Similarly, Wu et al. developed an amplification-free, SERS microfluidic approach for the detection of KRAS gene, commonly known for its mutations in cancer cells [84]. Based on their initial results, a subsequent study was performed for the development of a SERS biochip to profile multiplex mutational patterns in the DNA of cancer patients [85]. Moreover, Wang et al. developed an optofluidic metasurface-based biosensor for the detection of the ErbB2 gene, a well-established breast cancer biomarker. Their biosensor involved the use of a two-dimensional periodic array of silicon nanoposts-based technique that measures the refractive index change as a result of the target molecule immobilization [86].

Other studies employed the immunosensing strategy based on the detection of carcinoma antigen (CA) 125, as the most widely used glycoprotein for the early diagnosis of ovarian cancer [87,88]. On the one hand, Nami et al. developed a paper-based immunosensor by depositing silver nanoparticles onto reduced graphene oxide sheets and subsequently attaching cysteamine-functionalized gold nanoparticles that will further bond with the CA 125 antibody [87]. On the other hand, Nunna et al. developed a self-assembled monolayer by treating gold electrodes with thiourea for the subsequent attachment of the CA 125 antibody through the amine groups [88]. Similarly, Zheng et al. immobilized silver nanoparticles onto the surface of the microchannel for creating a SERS-active substrate that would allow for the attachment of antibodies to capture the target biomarkers CA 125, CA 153 simultaneously, and a carcinoembryonic antigen for breast cancer diagnosis [89]. Another study performed by Gao et al. investigated the efficiency of a SERS-based microfluidic biosensor to detect the prostate-specific antigen using magnetic beads and specific antibodies [90]. Furthermore, Guo et al. grew zinc oxide nanowires in parallel microfluidic channels for detecting the human α-fetoprotein and carcinoembryonic antigen, achieving a limit of detection of 1 pg/mL and 100 fg/mL, respectively [91].

### 3.2. Microbial Infection Diagnosis

BoC systems have also proved to be highly efficient in detecting microorganism-related infections, including bacterial, viral, and parasitic infections. In this context, a literature survey using the Scopus database and the keywords “microfluidic (bio)sensor” and “infection diagnosis” resulted in the identification of 10 papers published in 2018. All papers are summarized in Table 2 and described in the following paragraphs.

First, the identified papers investigated the detection efficiency of microfluidic biosensors towards *Escherichia coli*, *Bacillus subtilis*, *Pseudomonas aeruginosa*, *Staphylococcus aureus*, *Salmonella typhimurium*, and *Yersinia pestis* bacterial strains. Specifically, Narang et al. reported developing a microfluidic chip resting onto a resonator that emits an electrical signal further analyzed through a vector network analyzer. This device allowed for the real-time detection of *Escherichia coli* concentration and proliferation [100]. Furthermore, He et al. fabricated a paper-based microfluidic device for detecting *Escherichia coli* bacteria and subsequently its susceptibility towards a variety of antibiotics, including amoxicillin, ciprofloxacin, gentamicin, and nitrofurantoin, through observable color changes. Results indicated the potential of the device for diagnosing bacterial infections and providing the means for accurate antibiotic prescribing [101]. Additionally, Li et al. developed a droplet-based microfluidic platform containing a fluorescence-producing β-lactamase sensor (Fluorocillin sensor) for the detection and quantification of antibiotic-resistant bacteria [102]. By contrast, Lee et al. proposed a microfluidic platform employing redox-active gold nanoparticles conjugated with active electrochemical molecules and specific antibodies for subsequent attachment onto the bacteria surface. Their results showed a limit of detection of 10 CFU/mL and a dynamic range of 10–105 CFU/mL, thus holding great potential in bacteremia diagnosis [103]. Furthermore, two nanotechnology-enhanced strategies for the detection of *Salmonella typhimurium* were reported in the literature. On the one hand, Savas et al. reported the use of antibody- and DNA-based biosensors integrated within a fully-automated custom-designed microfluidic device employing gold nanoparticles for increasing sensitivity [104].

On the other hand, Thiha et al. reported the application of amine-ended aptamers immobilized onto suspended carbon nanowire surface integrated within a microfluidic chip [105]. Finally, Liu et al. investigated the efficiency of a microcantilever biosensor for the detection of *Yersinia pestis* using a microfluidic platform. In their study, the surface of the microcantilevers was functionalized for the subsequent attachment of two types of *Yersinia*-specific antibodies [106].

Second, the identified papers investigated the detection efficiency of microfluidic biosensors towards human adenovirus, and hepatitis B virus. On one hand, Jin et al. fabricated a novel microfluidic approach for both the isolation of viral DNA and its subsequent detection using an optical biosensor. In this manner, as sample quality is critical in infectious disease diagnosis, this method ensures a standardized and accurate sample preparation, resulting in a high quality and quantity of the viral DNA extracted [107]. On the other hand, Srisomwat et al. reported the development of a pop-up label-free electrochemical paper-based microfluidic device designed for DNA sensing from extracted DNA samples [108].

Ruiz-Vega et al. reported the development of an electrochemical device for the fast quantitative diagnosis of malaria. Using magnetic beads, their strategy allows for a single-step magneto-immunosensing through a single-use microfluidic paper double-sided screen-printed carbon electrode [109].

### 3.3. Neurodegenerative Disorder Diagnosis

Recent years have witnessed an increasing number of studies regarding the identification of neurodegeneration-related biomarkers [110,111,112]. In this regard, various biomarkers including amyloid-β peptides, tau proteins, neurofilament light, for neuronal injury, neurogranin, BACE1, SNAP-25, and synaptotagmin for synaptic dysfunction and/or loss, sTREM2, YKL-40, interleukins, tumor necrosis factor α, and lactoferrin for neuroinflammation due to the activation of microglia and astrocytes, and clusterin for apoptosis [113,114,115,116,117,118,119,120,121,122,123,124,125,126,127,128,129,130,131,132]. There are many studies investigating the efficiency of biosensors for the identification of these biomarkers [133,134]. However, the integration of microfluidics within these biosensors could bring the advantages of low sample and reagent consumption, high sensitivity and specificity, and low detection limit. However, the application of microfluidic biosensors for early diagnosis is still limited.

The literature survey using the Scopus database and the keywords “microfluidic (bio)sensor” and “neurodegenerative disorder diagnosis”, “neurodegeneration diagnosis”, “Alzheimer diagnosis”, “Parkinson diagnosis”, or “Huntington diagnosis” resulted in the identification of only one paper published since 2018.

Specifically, Ricci et al. developed a label-free biosensor using electrolyte-gated organic field-effect transistors for the electronic transduction for the identification of α-synuclein, a small protein known to be implicated in a series of neurodegenerative disorders, including Parkinson’s disease, Alzheimer’s disease, dementia with Lewy bodies, and multiple system atrophy [135,136,137,138]. The monoclonal anti-α-synuclein antibody was grafted on the gate electrode via two methods, namely Protein G and self-assembled monolayers. The integration of the biosensor within the microfluidic device has provided a sensitivity of up to 37(±5) mV/dec and a limit-of-detection of 0.25 pM [139].

Moreover, Song et al. developed a label-free nanosensor incorporating a nanopore layer consisting of an anodic aluminum oxide embedded in a nanostructured Fabry-Perot interference cavity, which serves as the sensing element for the detection of amyloid-β 42 and total-tau biomarkers. Specifically, the white light reflected from the nanosensor forms interference fringes that are further utilized as transducing signals. The efficiency and specificity of detection have proved to be up to 7.8 pg/mL of Aβ42 and 15.6 pg/mL of T-au in buffer [140]. Furthermore, Liu et al. fabricated a nanosensor for the highly sensitive and selective monitorization of extracellular potassium levels within the brain, a potential indicator for neurological disorders, including Alzheimer’s disease or epilepsy [141,142]. The optical readout is achieved by incorporating commercially available K^+^ indicators into mesoporous silica nanoparticles [142].

Although the number of studies is considerably limited, there is a great potential of microfluidic biosensors for the early and accurate diagnosis of neurodegenerative disorders, thus avoiding a great burden on the health systems [133,143].

## 4. Conclusions and Future Perspectives

BoC systems are based on micro-total analysis systems, which aim to provide complete analytical microscaled systems, thus allowing for the performance of standard laboratory functions, including sample injection, mixing, reaction, separation, and enrichment, and analyte detection. Specifically, these systems integrate biosensors with microfluidic technologies, resulting in higher detection sensitivity and specificity, resolution, automation, throughput, reproducibility, reliability, and accuracy. Therefore, the emergence of BoC systems has led to substantial advancements in PoC diagnostic applications that could bring significant improvements, especially in the resource-limited environment of the developing countries. It has brought diagnostics out of central laboratories to the patient bedside. While there are many breakthrough studies regarding microfluidic biosensors’ application for cancer and infectious disease diagnosis, neurodegenerative disorder diagnostics has not yet benefitted from these technologies. Since recent years have witnessed tremendous advancements in the neurodegenerative biomarker arena, future perspectives should focus on the development of BoC systems for diagnosing such disorders.

## Figures and Tables

**Table 1:** A summary of the identified papers investigating the use of microfluidic biosensors for cancer diagnosis.

Biomarker Type	Biosensor Type	Measuring Principle	Target	Capture Molecule	Cancer Model	Limit of Detection	Ref
CTCs	immunosensor	fluorometry	EpCAM	EpCAM antibody	MCF-7 breast cancer cells	n.r.	[74]
	immunosensor	fluorometry	EpCAM	EpCAM antibody	EJ138, HT1376, HT1197, and RT4 human bladder cancer cells	n.r.	[75]
	aptasensor	electrochemical impedance	A549 cells	DNA aptamer	A549 human lung carcinoma cells	1.5 × 10^4^ cells/mL	[76]
	immunosensor	electrochemical impedance	MC1R	MC1R antibody	SK-MEL-2 human melanoma cells	10 cells/10 mL	[77]
miRNA	aptasensor	fluorometry	miRNA-1246-TAMRA	3-aminopropyltrimethoxysilane	non-small cell lung cancer	n.r.	[78]
	aptasensor	amperometry	miRNA-197	complementary single-stranded DNA	-	1.28 nM	[79]
	aptasensor	colorimetry	miRNA-21	complementary single-stranded DNA	-	4.1 pM	[80]
	aptasensor	fluorometry	miRNA-21	thiol-modified hairpin DNA probe	MCF-7 breast cancer cells	0.0033 fM	[81]
	enzyme-based sensor	chronoamperometry	miRNA-19b and miRNA-20a	Cas13a effector	brain cancer	10 pM	[82]
DNA	aptasensor	chronoamperometry	BRCA1, BRCA2, and p53 breast cancer genes	thiolated 19-mer BRCA1, 17-mer BRCA2, and 17-mer p53	breast cancer	-	[83]
	aptasensor	SERS	KRAS gene	molecular beacon probes	MDA-MB-435 and SW480	50 fM	[84]
	aptasensor	SERS	KRAS gene	molecular beacon probes	colorectal cancer	10 fM	[85]
	immunosensor	reflectometry	ErbB2 gene	anti-ErbB2 antibody	breast cancer	0.7 ng/mL	[86]
proteins	immunosensor	chronoamperometry	CA 125	CA 125 antibody	ovarian cancer	0.78 U/mL	[87]
	immunosensor	capacitance measurement	CA 125	CA 125 antibody	ovarian cancer	-	[88]
	immunosensor	SERS	CA 125, CA 153, carcinoembryonic antigen	CA 125, CA 153, carcinoembryonic antigen antibodies	breast cancer	0.0001 U/mL	[89]
	immunosensor	SERS	prostate specific antigen	prostate specific antigen antibody	prostate cancer	0.01 ng/mL	[90]
	immunosensor	fluorometry	α-fetoprotein and carcinoembryonic antigen	α-fetoprotein and carcinoembryonic antibodies	-	1 pg/mL and 100 fg/mL	[91]

n.r.–not reported in the paper.

**Table 2:** A summary of the identified papers investigating the use of microfluidic biosensors for microbial infection diagnosis.

Microbial Type	Microbial Strain	Biosensor Type	Measuring Principle	Target	Capture Molecule	Limit of Detection	Ref
bacteria	*Escherichia coli*	microwave-based sensor	vector network analysis	*Escherichia coli*	-	n.r.	[100]
	*Escherichia coli*	optical sensor	colorimetry	*Escherichia coli*	-	n.r.	[101]
	ampicillin-resistant *Escherichia coli,* ampicillin-susceptible *Escherichia coli*, and *Bacillus subtilis*	enzyme-based sensor	fluorometry	β-lactamase	-	1 × 10^4^ cells per well	[102]
	*Pseudomonas aeruginosa* and *Staphylococcus aureus*	immunosensor	amperometry	*Pseudomonas aeruginosa* and *Staphylococcus aureus*	mouse anti-*Pseudomonas aeruginosa* antibody and mouse anti-*Staphylococcus aureus* antibody	10 CFU/mL	[103]
	*Salmonella typhimurium*	immunosensor and aptasensor	amperometry	*Salmonella typhimurium* and target DNA	anti-*Salmonella* antibody and DNA probe	2.7 × 10^1^ CFU/mL and 0.94 nM	[104]
	*Salmonella typhimurium*	aptasensor	chemiresistive detection	-	amine-ended aptamers	10 CFU/mL	[105]
	*Yersinia pestis*	immunosensor	microcantilever detection	*Yersinia pestis*	*Yersinia*-specific antibodies	n.r.	[106]
viruses	human adenovirus	aptasensor	fluorimetry	extracted viral DNA	DNA probe	10 virus copies	[107]
	hepatitis B virus	aptasensor	differential pulse voltammetry	target DNA	DNA probe	1.45 pM	[108]
parasites	*Plasmodium falciparum*	immunosensor	magnetometry	*Plasmodium falciparum* lactate dehydrogenase	Anti- *Plasmodium* lactate dehydrogenase monoclonal capture and c-MAb and bd-MAb detection antibodies	200 ng/mL	[109]

n.r.–not reported in the paper.
